# The Foreign Journals: Medical Jurisprudence

**Published:** 1839-07

**Authors:** 


### MEDICAL JURISPRUDENCE.

### *Case of alleged Poisoning by the Sulphocyanic and Hydrocyanic Acids.* By Dr. Graff, of Darmstadt.

A mechanic, who had gone home the previous evening apparently in good health, was found the following morning lying dead in bed, with the door of the room and the windows fastened. A medico-legal inspection of the body was required to be made, and this accordingly took place about 24 hours after death.

On a table in the room was found a small glass retort with its body broken. It contained yellow-coloured crystals, afterwards proved to be the ferrocyanate of potash, mixed with a gray and blue-coloured matter. Near it was some strong sulphuric acid in a bottle; and in another small bottle, which seemed to have been used as a receiver, was about a scruple of a clear yellow liquid. The deceased was dressed, and lying on the bed in a composed attitude. The countenance was pale and tranquil: a mucous frothy liquid, tinged with blood, issued from the nostrils. A strong and offensive smell exhaled from the body, but there was no odour of hydrocyanic acid mixed with it. The skin was generally in a state of horripilation. On opening the cranium, the only appearance observed was congestion of the vessels of the brain. The mouth and fauces were free from excoriation and inflammation: but the mucous membrane of the lower part of the trachea was inflamed, and of a deep red colour. The lungs were congested and dark coloured. Dark blood was contained in both sides of the heart, but especially on the right side. Coagula were found in both cavities, as well as in the aorta and venæ cavæ. The stomach, having been included between ligatures, was removed. Externally, the vessels near the pylorus appeared congested; but at the greater end there were several dark-coloured patches. It was then opened, and the contents reserved for analysis. The mucous membrane was pale, without inflammation or excoriation: but at the fundus, for about three square inches, it was of a dark brownish black colour, softened and pulpy: there was no sign of inflammation beneath or around this discoloured and softened membrane. Some half-digested food was mixed with the contents. The bladder was much distended with urine, the other viscera presented nothing abnormal. The only other appearances remarked were that the nails of the fingers were of a deep violet hue; and the penis, when the body was first seen, was in a state of semi-erection. There was not the least odour of prussic acid in any of the cavities or organs.

From these facts the following conclusions were derived:

1. Death was the result of suicide. This was established by the circumstantial evidence. It was ascertained that the deceased had some knowledge of chemistry. He had made no secret of his intention to destroy himself, and had repeatedly enquired of friends respecting the best method of preparing prussic acid.

2. Death must have taken place at night between ten and eleven o'clock, since the deceased was seen to sup with his accustomed appetite at eight in the evening; and the half-digested state of the contents of the stomach, seems to show that he had not survived his evening meal longer than two or three hours. [It is a remarkable omission in the report, that, although the appearances of the body, &c., are marked down in no less than 104 sections, not a word is said concerning the presence or absence of *warmth* or *rigidity* in the body when first discovered; conditions which, properly observed, throw considerable light on these important questions of survivorship.]

3. Death was most probably due to the action of a powerful narcotic, mixed with some acrid or corrosive poison. The post-mortem appearances establish this. (?) The absence of erosion in the mouth and fauces shows that the corrosive poison could not have been very concentrated. Any of the strong mineral acids could not, therefore, be suspected.

4. Death took place rapidly. This was proved by the fact, that there was not the least sign of redness or irritation around the spot in the stomach, which had become corroded. Death did not result from this corrosive action, but from the influence exerted by the poison on the brain and nervous system. The position of the body seemed to show that death was not preceded by pain.

5. The poison was doubtless a mixture of the sulphocyanic and prussic acids, probably prepared and taken by the deceased immediately after its preparation. Very different statements are made relative to the appearances left by prussic acid in the body. Vogt says it produces redness of the mucous membrane of the alimentary canal and air-passages, and rapid putrefaction. Orfila denies that redness of the mucous membrane is a result of its action, and affirms that the body is a long time before it putrefies. In a case reported in Hufeland's journal, the blood had a smell of bitter almonds, the cerebral vessels were congested, and the stomach was much inflamed and softened. The blood was liquid, and thick like oil. In a case of Casper's, the only appearances were, a strong odour of the acid, and the blood of a dark violet colour. In this case, putrefaction took place slowly; but the absence of all odour of prussic acid, and the corroded state of the stomach, rendered it unlike the recorded cases of poisoning by that liquid. The most-carefully conducted experiments by Dr. Merk did not lead to the detection of this poison, in the contents of the mouth, œsophagus, stomach, or intestines; but he thought he discovered a faint trace in the yellow liquid, contained in the receiver already mentioned. This, however, was too faint to allow an opinion to be expressed with any medico-legal certainty. By pursuing the process supposed to have been adopted by the deceased, namely, the distilling of sulphuric acid with ferrocyanate of potash, Dr. Merk obtained principally sulphocyanic acid mixed with a small quantity of prussic, formic, and sulphurous acids. As sulphocyanic acid is a corrosive and deadly poison (?) without smell, the whole of the facts connected with the death of the deceased were considered to be at once explained by his having prepared and swallowed this substance. Three drops of the liquid obtained by Dr. Merk, killed a young rabbit in half a minute! and on inspection, the appearances very closely resembled those found in the body of the deceased. On the other hand, Prof. Liebig, of Giessen, was requested to determine what products were obtained on distilling sulphuric acid and ferrocyanate of potash. He procured no sulphocyanic acid, but simply formic and sulphurous acids. Moldenhauer, another chemist, on performing the experiment obtained this acid, mixed with the others mentioned. The contents of the recipient found on the table, in the deceased's room gave, on analysis, sulphuric acid, ferrocyanate of potash, and traces of sulphocyanic acid. (?) The contents of the deceased's stomach, amounting to sixteen ounces, were divided into two equal portions. One half, after phosphoric acid had been added, was distilled into a receiver containing a solution of pure potash. A persalt of iron was added to the alkaline solution after the distillation had gone on sufficiently, but the test did not indicate the presence of either of the suspected poisons. The other half was distilled into a receiver, containing the ammoniaco-nitrate of silver: but this, on subsequent examination, gave only the faintest traces of what was supposed to be cyanide of silver. The conclusion came to was that sulphocyanic acid, mixed with a small portion of the other acids already mentioned, had destroyed life.

*Henke's Zeitschrift*, 1838.

[Remarks.—The interest of this case is in great part lost, from its not being at all clearly made out, what poison the deceased really took. Minute chemical details occupy about one half of the report; but they leave the case in greater mystery, than they found it. The case is so far important, that if it really be one of poisoning by the sulphocyanic acid, it is the first on record, and is much opposed to the generally received opinions of toxicologists, for it is here represented to be as fearfully destructive as hydrocyanic acid itself. The experiments of Prof. Meyer, of Bonn, have, however, shown that this acid has but very little energy as a poison. Three drachms of the pure acid, in three doses, had no effect when introduced into the stomach of a rabbit; while we are told by Dr. Graff, in this paper, that *three drops* killed a rabbit in *half a minute*. How can such an extraordinary discrepancy be reconciled?—We think by showing that Dr. Graff was in error, supposing that in his case the animal was not killed by suffocation, a very common source of failure in experimenting with poisons on rabbits.

In the case reported, Dr. G. seems to have adopted the idea that prussic acid could not have been taken, because there was no odour on inspection, or in the apartment; a fact, however, wholly insufficient to justify the inference, since the acid may be taken in a small but still a poisonous dose; and after twenty-four hours' exposure, or even less, it may not be indicated by any smell issuing from the body. The corroded state of the stomach was considered another proof of this poison not having been taken: but this might have proceeded from some corrosive substance having been swallowed simultaneously with prussic acid. At any rate, the sulphocyanic acid, to which this effect is assigned by the reporter, although an irritant, is not a corrosive; and its action on cork, referred to by him as evidence of its possessing this property, is really due to another cause; namely, to its combining with the minute traces of iron contained in that substance. But further:—Is sulphocyanic acid a product of the distillation of ferrocyanate of potash and sulphuric acid? We confess we do not see how this can happen, and we would rather abide by the results obtained by Liebig than trust to those of the other chemists referred to by the author, who seem to have undertaken the investigation with the full intention of obtaining sulphocyanic acid. The details of the experiments of the latter chemists are by no means satisfactory; and considering that sulphocyanic acid may be readily detected in an infinitesimal proportion, our impression is that they obtained none, or but the faintest traces. On the other hand, prussic acid is known to be a product of this distillation, when the sulphuric acid is diluted; and also, but perhaps to a less extent, when the acid is concentrated. It appears to us that this was the poison which the deceased really prepared and swallowed, probably mixed with sulphuric acid. Our opinion is founded on these facts: he must have died quickly; there was no sign of vomiting or purging; there were no marks of irritation or inflammation in the alimentary canal; and he had materials at hand from which prussic acid might be made, with sufficient chemical knowledge for its preparation. The non-discovery of any traces of it in the contents of the stomach might be accounted for.

To test the correctness of the preceding views, we distilled equal parts of ferrocyanate of potash and strong sulphuric acid to dryness. A small quantity of a yellowish coloured liquid was obtained in the receiver, in which the odour of hydrocyanic acid was plainly perceptible. Nitrate of silver gave a white, curdly precipitate, showing the presence of hydrocyanic or sulphocyanic acid, or both. The liquid was now divided into two parts:—To one, a few drops of sesquichloride of iron was added, and a slight reddish tinge was produced, which disappeared on adding chloride of gold. This showed the presence of the faintest traces of sulphocyanic acid. To the other portion of liquid, a saturated solution of protosulphate of iron was added. By the subsequent addition of potash and dilute sulphuric acid, a comparatively large proportion of Prussian blue was left; thus proving that the main constituent of the distilled liquid was really *hydrocyanic acid.* The residue in the retort, which was dry and hard when dissolved in water, yielded a considerable quantity of Prussian blue, and the liquid chiefly contained sulphate of potash. We consider that the facts of the case thus receive an explanation, and we see no reason therefore for admitting that it forms an exception to the general experience of toxicologists. The sulphocyanic acid, if obtained at all by the deceased, must have been in too small a quantity to have led to fatal consequences within so short a period. Its operation, besides, is that of an irritant, not of a narcotic.]

